# An improved defocusing adaptive style transfer method based on a stroke pyramid

**DOI:** 10.1371/journal.pone.0284742

**Published:** 2023-04-24

**Authors:** Jianfang Cao, Zeyu Chen, Mengyan Jin, Yun Tian

**Affiliations:** 1 Department of Computer Science & Technology, Xinzhou Normal University, Xinzhou, China; 2 School of Computer Science & Technology, Taiyuan University of Science and Technology, Taiyuan, China; Menoufia University, EGYPT

## Abstract

Image style transfer aims to assign a specified artist’s style to a real image. However, most existing methods cannot generate textures of various thicknesses due to the rich semantic information of the input image. The image loses some semantic information through style transfer with a uniform stroke size. To address the above problems, we propose an improved multi-stroke defocus adaptive style transfer framework based on a stroke pyramid, which mainly fuses various stroke sizes in the image spatial dimension to enhance the image content interpretability. We expand the receptive field of each branch and then fuse the features generated by the multiple branches based on defocus degree. Finally, we add an additional loss term to enhance the structural features of the generated image. The proposed model is trained using the Common Objects in Context (COCO) and Synthetic Depth of Field (SYNDOF) datasets, and the peak signal-to-noise ratio (PSNR) and structural similarity index (SSIM) are used to evaluate the overall quality of the output image and its structural similarity with the content image, respectively. To validate the feasibility of the proposed algorithm, we compare the average PSNR and SSIM values of the output of the modified model and those of the original model. The experimental results show that the modified model improves the PSNR and SSIM values of the outputs by 1.43 and 0.12 on average, respectively. Compared with the single-stroke style transfer method, the framework proposed in this study improves the readability of the output images with more abundant visual expression.

## 1. Introduction

Artificial intelligence (AI) has attracted considerable attention due to its powerful capacity in multiple application fields, such as intelligent processing and self-adaptive learning. AI can handle vast quantities of data and solve complex questions through algorithm and model training and optimization. AI is also equipped with intelligent self-learning, which enables it to gradually improve its self-learning and judgment capacities based on data and experience accumulation and analysis. These virtues guarantee its cross integration in multiple fields. With the rapid development of AI technology represented by deep learning, an increasing number of fields have begun to cross integrate, such as groundwater storage modeling [1], climate change forecasting [2], environmental factor analysis [3], air pollution prediction [4], satellite image classification [5], weed detection [6], forest area classification [7], agricultural water resource management [8], biomedical fields [9–11] and biometric identification [12]. These studies have brought great convenience and improvement to people’s lives and work. Therefore, AI has attracted considerable attention in the current scientific and technological fields and has great development prospects.

Style transfer is also a typical practice of integrating art and deep learning technology. Specifically, given a content graph and a target style graph, the purpose of style transfer is to transform the style of the original content graph into the style of the target graph while ensuring that the semantic information of the original content is not lost. At present, all kinds of drawing software, represented by mobile phone image applications, use a large number of related technologies to provide people with leisure and entertainment functions. Inspired by convolutional neural networks (CNNs), Gatys et al. [13] first studied how CNNs can be used to recreate pictures in natural famous painting styles. They suggested modeling photo content as feature responses from a pretrained CNN, and others have modeled art styles as summary feature statistics. Subsequently, the model-based iterative optimization method proposed by scholars improved t image stylization efficiency. Li et al. [14] proposed replacing the Gram matrix matching in the Gatys model with the Markov regularization model by combining the Markov random field and a VGG network. Johnson et al. [15] and Ulyanov et al. [16] proposed a fast neural style transfer model that achieves real-time stylization by training forward neural networks. Although the above generation model method is two orders of magnitude faster than the previous style transfer method based on image iteration, it can generate images of only specific styles. To obtain other styles, a feedforward generation network needs to be retrained, so it is less flexible and very time consuming. Therefore, the single model multistyle generation network began to appear, which integrates multiple styles into one model to improve the efficiency of the feedforward network. Zhang et al. [17] proposed the concept of the CoMatch layer, which requires the model to learn multiple styles and then use the target style image features as a signal input to guide the input image to match the style features in the CoMatch layer to achieve style transfer. He et al. [18] proposed the deep-exemplar-based method. By using a large sample database in combination with the input reference image, a style transfer scheme is generated after comprehensive calculation. Li et al. [19] proposed a style selection model containing multiple styles, which uses image pixels as a signal input to control stylized image generation. This model can synthesize more than 300 textures and can generate 16 styles of feedforward networks. The above method is more flexible, but a single network can achieve only limited categories of style conversion. Chen et al. [20] proposed using a method called "style swap" to achieve any style transfer model. The method uses a pretrained VGG network to extract content and style features from the input information, divides them into activation blocks of the same size, and then matches each content activation block with the most similar style activation block to obtain a generated image. The model has high flexibility, but the stylization speed is slow. Huang et al. [21] proposed a method called the adaptive instance normalization (AdaIn) layer. The algorithm calculates the mean and variance in the extracted content features and style features according to the channel dimension, applies the mean and variance in the style features to the content features, and reconstructs the resulting image through the decoder. Li et al. [22] introduced the whiten-color transformation (WCT) mechanism into the style transfer algorithm. This method stripped the original color information of the content features and then used the color information of the style features to achieve the transfer effect. Park et al. [23] proposed a self-attention network (SANet), which uses a self-attention mechanism to mix content features and style features and then flexibly modifies local styles according to the semantic spatial distribution of the content images. In addition, some methods add a ’stroke control’ function to the original style transfer algorithm. A single model can generate not only good quality style transfer results but also control the thickness of textures in the image, thus providing users with more choices. Jing et al. [24] proposed a network structure called the stroke pyramid. By limiting the correspondence between the stroke size and the style image size, the algorithm uses convolutional layers with different receptive fields as different stroke branches to achieve continuous stroke size control in the spatial dimension. Yao et al. [25] proposed an arbitrary style transfer architecture with multi-stroke fusion. The algorithm uses multi-scale style swaps to generate multiple stroke features and then fuses each stroke feature through self-attention maps to achieve adaptive coordination of style and content in the spatial dimension. Recently, an increasing number of reports on the cross-fusion between style transfer algorithms and classic models have emerged. Zhang et al. [26] proposed a hierarchical vision transformer using strip window attention. This approach realizes accurate style transfer by focusing on local image domains and adapting to a wide range of styles. Feng et al. [27] utilized a synthesized transformer-based automatic encoder for style transfer. This approach combines an optimization strategy to learn the style and content representations contained in images to exert accurate control over the style transmission process.

However, most existing algorithms focus only on generating images with similar styles, ignoring the destruction of image semantics in the style transfer process. For the feedforward network model trained for style images with many lines and dense textures, the saliency of the texture-expressing style in the generated image is higher than that of the texture-expressing content, thus masking the content information to be expressed by the image, making it impossible or difficult for readers to interpret the content in the image. Inspired by the gold tower of strokes, we use strokes of various sizes to depict the generated images to enhance the semantic saliency of the images. We use the defocus estimation algorithm to predict the clarity of the image in pixels to obtain the distribution of clear areas. The clear region is usually the content focus of the image, which contains rich semantic information. In contrast, the fuzzy region expresses less semantic information. We use smaller strokes to depict the clear areas, that is, to retain as many detailed features as possible contained in the original image, and use larger strokes to depict blurred areas to distinguish them from clear areas, increasing the saliency of the main image content. Additionally, we improve the network structure of the stroke gold tower. The receptive field of each branch of the original stroke pyramid is small, and it cannot accommodate large geometric microstructures during the training process, resulting in a reduced style effect. Therefore, we use dilated convolution to make each branch obtain a larger receptive field. To address the problem of jagged traces caused by the superposition of multiple dilated convolutions, we use various sizes of dilated convolution kernels. The defocus blur estimation algorithm is a method used to predict the blur degree of each pixel in a real image. The traditional method is to generate a complete defocus map by iteration. Zhuo et al. [28] and Karaali et al. [29] calculated the ratio between the gradient of the input image and the re-blurred image as the blurring amount of the edge position; then, they propagated the blurring amount of the edge to the adjacent pixels by an iterative method to calculate the complete defocus image. Shi et al. [30] used a sparse fuzzy scale instead of a fuzzy quantity to represent the blur degree of pixels. Tang et al. [31] used spectral amplitude as a clue to estimate the blur amount and estimated the blur amount at these edge locations by establishing the relationship between the amount of spatially varying defocus blur and spectrum contrast at edge locations. Xu et al. [32] divided the image into local image blocks to calculate the blur amount and to improve prediction efficiency. Park et al. [33] proposed a multi-scale image block extraction strategy to reduce the algorithm dependence on the image block size, thereby improving the synthesis result quality. However, the iterative algorithm is time consuming, and the defocus estimation algorithm based on deep learning was proposed. Lee et al. [34] integrated the Synthetic Depth-of-Field (SYNDOF) dataset, constructed a decoder corresponding to VGG-Net and introduced the domain adaptation method to improve the defocus estimation efficiency. We fuse the feature results output by the multiple branches and the defocused image according to the clarity of the spatial dimension so that the algorithm can weigh the size of the stroke according to the richness of the semantic information expressed by the image.

In this paper, we propose an improved multi-stroke defocus adaptive style transfer method based on a stroke pyramid. The main contributions include the following aspects.

1. A depth estimation based on defocus blur is introduced, and the feature maps of various stroke sizes are combined by using the feature weights after soft threshold segmentation. The framework of this paper can adaptively adjust the strokes and textures of the output image according to the blur degree of the image. The difference between the defocused blurred area and the focused clear area after stylization is more obvious. The main body of the image can be better expressed and has richer textures.
2. By using residual blocks composed of dilated convolutions to expand the receptive field of each stroke pyramid branch, the network can learn to depict larger strokes.
3. Gram loss and mean standard deviation loss are used as the evaluation criteria for style similarity so that the generated image can better retain the structural information from the style image.

This article is divided into three parts. In the following section, we introduce the theoretical basis of the algorithm, network structure, loss function calculation and the experiment performed in this study. In the third section, we focus on the results obtained in this study, and comparisons are also made between our model and those reported in the literature. In the last section, we summarize and discuss the results of this study and propose future research directions for style transfer algorithms.

## 2. Methodology

### 2.1 Background theory

#### 2.1.1 MobileNetV3 feature extraction network

The MobileNetV3 model is a 20-layer convolutional neural network for extracting features, and part of its structure is shown in Table 1. The network adds a squeeze-and-excitation (SE) network to the core architecture and improves the quality of the features extracted by the network by showing the interdependence between the convolutional feature channels in its modeling network.

**Table 1:** MobileNetV3 feature extraction network structure.

Input	Operator	outChannel	stride
224×224×3	conv2d	16	2
112×112×16	bneck_1_1, 3×3	16	1
112×112×16	bneck_1_2, 3×3	24	2
56×56×24	bneck_2_1, 3×3	24	1
56×56×24	bneck_2_2, 5×5	40	2
28×28×40	bneck_3_1, 5×5	40	1
28×28×40	bneck_3_2, 5×5	40	1
28×28×40	bneck_3_3, 3×3	80	2

The SE mechanism automatically acquires the importance of each feature channel by learning and then, based on those results, enhances the useful features while it suppresses features that are not useful for the current task; the network can learn to selectively emphasize or suppress certain feature information by using global information. Due to the need for different processing of image feature information, this paper uses the pretrained MobileNetV3 to realize the feature extraction operation of the generated network. Compared with the VGG feature extraction network, MobileNetV3 has fewer convolution layers and network parameters, making it more lightweight, thus reducing the calculations required of the network. In this paper, we use the output of the bneck_2_1 layer as image feature information. Compared with the original image size, this feature is twice as small in size, containing only 24 channels. The smaller number of channels ensures the weight of the subsequent network. Different subsequent processing branches can expand the channels as required to improve the network performance.

#### 2.1.2 Stroke pyramid

The stroke pyramid was first proposed by Jing et al. [24], who corresponded style images of different sizes to strokes of different sizes for network training. Although two images generated with two different strokes show the same semantics and style, they have extensive and delicate differences in texture structure. In the style transfer algorithm, the style similarity between the style image and the output image is calculated by the Gram matrix. The Gram matrix is sensitive to the size of strokes, and therefore, a change in the size of the style image is equivalent to a change in the stroke size of the image; thus, the trained generative network generates different size strokes.

**Fig 1:**
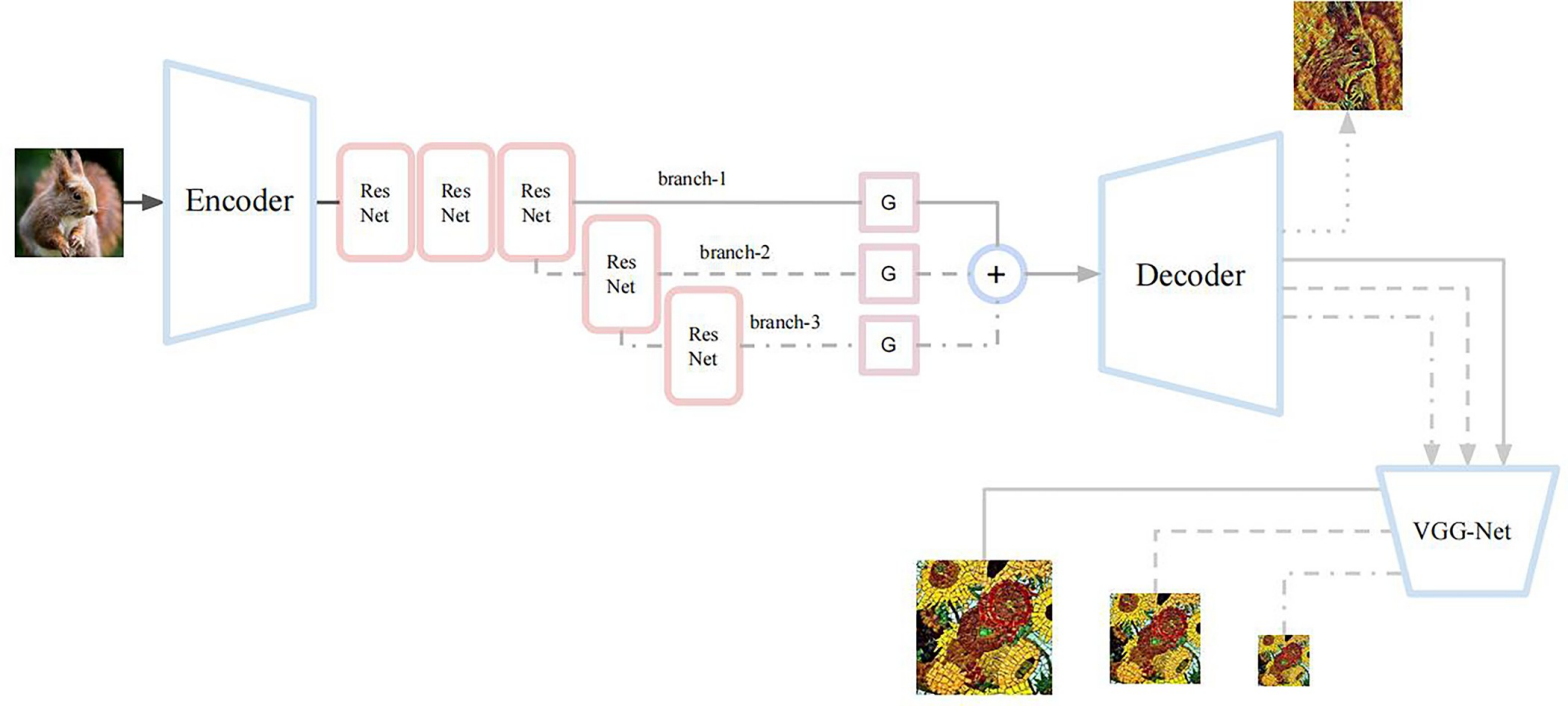
Stroke pyramid network structure.

By controlling the dependence between the receptive field of the generative network and the size of the style image, continuous multi-stroke style transfer in the spatial dimension is realized. As shown in Fig 1, the stroke pyramid network is divided into a decoder encoder, encoder decoder, stroke pyramid migration network, and VGG-Net loss network. The decoder consists of three convolutions, which are responsible for downsampling the input image to extract image features. The encoder is composed of one convolution and two deconvolutions and is responsible for reconstructing image features into output images. The shallow network has a smaller receptive field and is better at learning to draw small strokes. In contrast, a deep network with a larger receptive field is better at learning to draw large strokes. Since the output of each branch is the input of the previous branch, the output of the different branches has a certain correlation in space, which is conducive to the expression of multiple strokes in space.

### 2.2 Method description

**Fig 2:**
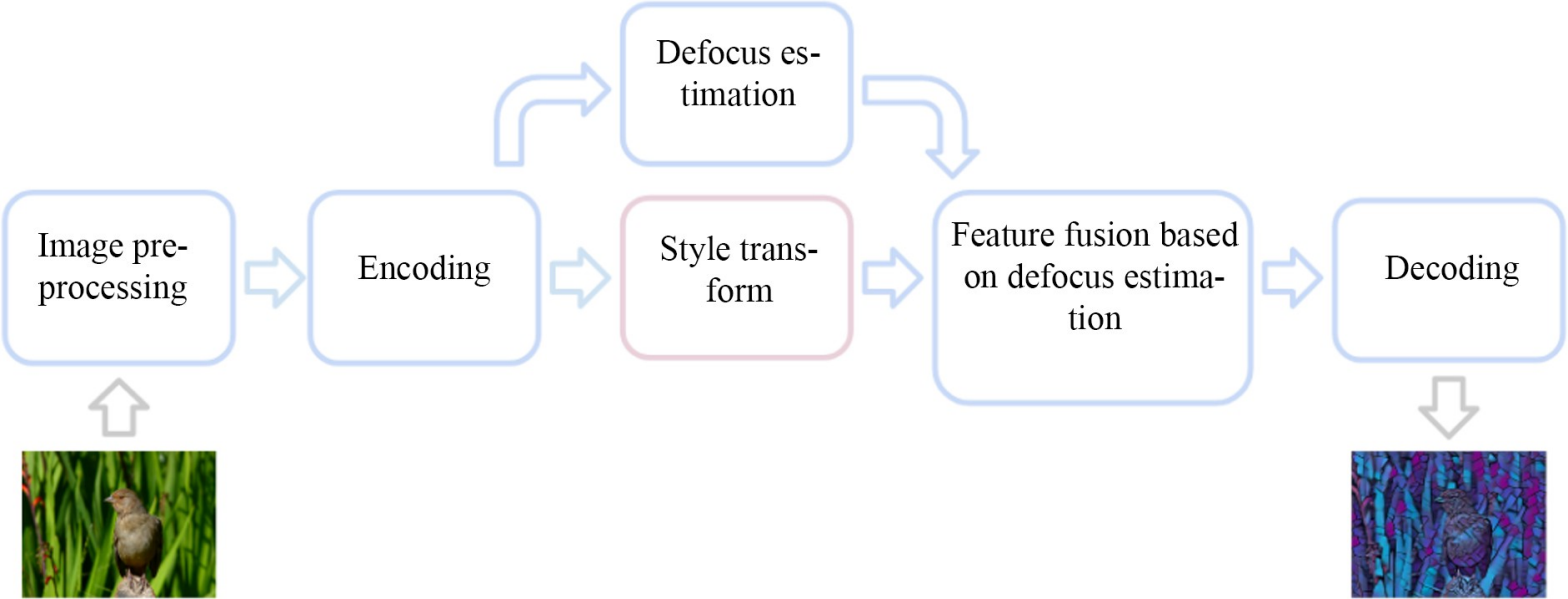
Workflow of the improved defocus adaptive style migration method based on the stroke pyramid.

The workflow of the improved defocus adaptive style migration method based on the stroke pyramid is shown in Fig 2. First, we preprocess the image that needs to be stylized, adjust the mean and standard deviation of the image in the channel dimension, and then extract the high-dimensional image features through an encoder and perform style migration and defocus estimation processing for the high-dimensional features. Finally, we fuse the stylized features based on defocus information and obtain the stylized image through decoding operations.

#### 2.2.1 Network overall design

**Fig 3:**
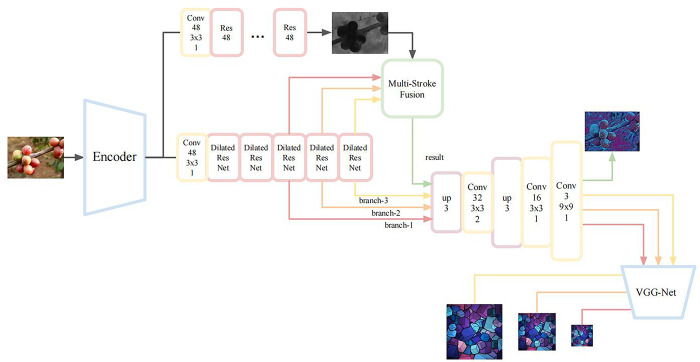
Defocusing adaptive multi-stroke network structure.

The defocusing adaptive multi-stroke network is shown in Fig 3. The design criterion of the model is to design a larger receptive field on each branch of the stroke pyramid so that the network can learn larger strokes while ensuring that the network layer is not too deep; a shallower network layer ensures the continuity of adjacent strokes in space. At the same time, defocus estimation is used to detect the distribution of clear and blurred areas in the image, and multiple strokes are fused with this as a reference to highlight the main area in the output image and to create a better migration effect.

The model structure shown in Fig 3 is comprises an encoder, a decoder, a style converter, a defocus blur estimation module, a soft mask gating module and a loss network. The encoder comprises the first four layers of MobileNet v3 and is responsible for encoding the image into a 24-channel high-dimensional feature map. Then, the high-dimensional feature maps are passed to the style converter and the defocus estimation module. The style converter can make the current feature obtain a specific style, which comprises several residual blocks with dilated convolution, and the number of channels in each residual block is 48. When considering the problem of generating the receptive field of the network, the first two residual blocks are used to improve the depth of the network, and the remaining residual blocks form independent branches. The features output by each branch skip the subsequent residual blocks and directly enter the decoder. Such structural division enables the different branches to extract feature maps under various receptive fields. The defocus blur estimation module converts the received image features into a single-channel defocus map that is 4 times smaller than the original image size. Each code value of the defocus map belongs to the interval [0,1], which represents the degree of blurring at this position. Then, the features of multiple branch outputs are fused with reference to the defocus map. The decoder consists of two upsampling layers and a convolution layer, which is responsible for reconstructing the feature map into a stylized image. Considering the checkerboard effect of deconvolution, nearest neighbor interpolation is used instead of deconvolution as the upsampling layer. When training the network, the output image, the style image, and the content image are transmitted to the VGG-19 network, and the extracted layer features are used to calculate the loss function.

#### 2.2.2 Improvement

(1) Introducing a hybrid dilated convolution module to expand the receptive field of the network

**Fig 4:**
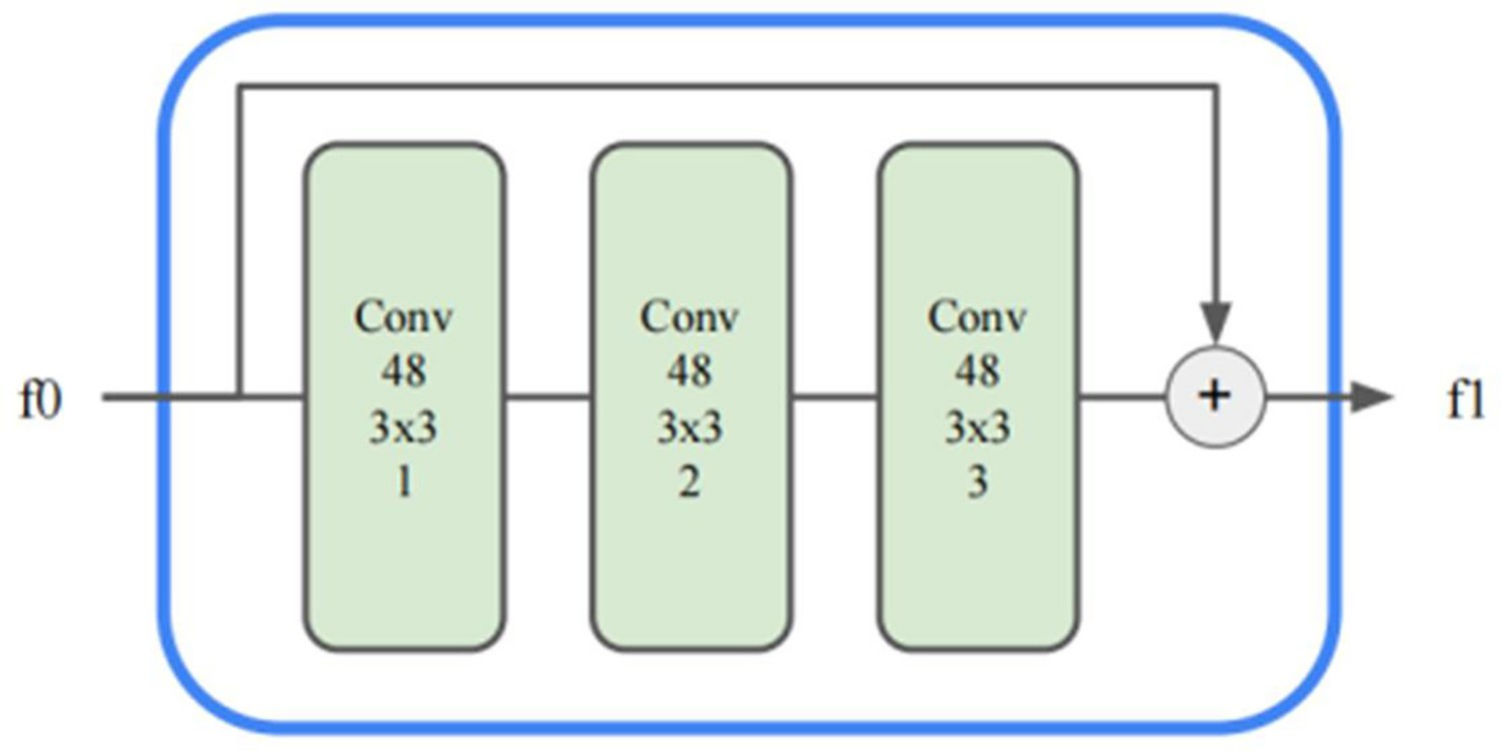
Structure of the hybrid dilated convolution module.

The stroke size increases as the size of the style image increases. When the receptive field of a branch in the generation network is smaller than that of its corresponding stroke size, the branch cannot fully draw the stroke and can learn to draw only a portion of the stroke in each region, thus affecting the quality of the style transfer. Therefore, for large stroke sizes, the network needs a larger receptive field to learn the global stroke configuration. To address this problem, we introduce a hybrid dilated convolution module to improve the receptive field of branches with different stroke sizes. The hybrid dilated convolution module is shown in Fig 4. The module comprises three convolution layers with the same convolution kernel size but different expansion rates. The expansion rates of the convolution layers are 1, 2 and 3 in order from front to back. Finally, the output features are added to the input features to obtain the final result.

Compared with that of the ordinary residual block composed of two convolution kernels of 3x3 size, the residual block composed of the dilated convolution module has a larger receptive field. Taking the convolution kernel with a size of K×K as an example and assuming that the number of channels C remains unchanged, calculating a single pixel in the output feature involves K*K*C pixels in the input feature, and the size of the area covered in the spatial dimension is K*K. The more pixels in the input features involved in a single pixel of the output feature, the larger the receptive field of the network. We take the size of the area covered by the associated pixel as the reference for the receptive field size and then compare the receptive field of a single branch before and after the improvement. The number of correlated pixels in the ordinary residual block is 5*5*C = 25*C, and the coverage area is 5*5. The number of associated pixels of the hybrid dilated convolutional residual network is 13*13*C = 169*C, and the size of the coverage area is 13*13. The results show that the hybrid dilated convolution module increases the receptive field of a single branch by more than 6 times the original approach and larger strokes are learned to improve the global effect. Moreover, the dilated convolution of such permutations and combinations makes the coverage more uniform while avoiding the checkerboard effect caused by the reuse of the associated pixels in the spatial dimension.

(2) Introducing the mean standard deviation loss to enhance the network sensitivity to shape features

Conventional style transfer algorithms use the Gram matrix as a method to measure style similarity. However, the Gram matrix is not sensitive to the shape structure in the style image spatial dimension. For the style image with no obvious color difference between adjacent regions, the Gram matrix has difficulty retaining the structural style image information, resulting in a small loss in the Gram matrix during the training process, but the effect of generating the image is very poor. In this paper, we introduce the mean standard deviation loss as an additional loss term in the style loss function. The calculation steps of the mean standard deviation loss are as follows: The style image and the output image are introduced into VGG-Net to extract features, the mean and standard deviation of the features are calculated in the spatial dimension, and the results are connected in the channel dimension. Finally, the MSE loss of the two is calculated.

The mean and standard deviation are calculated as follows:

$$\mu (F)=\frac{1}{HW}\sum_{h=1}^{H}\sum_{w=1}^{W}F_{hw}$$ (1)

$$\sigma (F)={\left[\frac{1}{HW}\overset{H}{\underset{h=1}{-}}\sum_{w=1}^{W}{\left(F_{hw}‐\mu (F)\right)}^{2}\right]}^{\frac{1}{2}}$$ (2)

$$L=\|\mu (F^{l}(I_{o}))-\mu (F^{l}(I_{s}))\|+\|\sigma (F^{l}(I_{o}))-\sigma (F^{l}(I_{s}))\|$$ (3)

where *I*_*o*_ represents the style image, *I*_*s*_ represents the output image, and *F*^*l*^(•) represents the first layer feature extracted by VGG-Net.

(3) Introducing defocus prediction and fusing multi-stroke features to enhance semantic information in generated images

We use a neural network to predict the defocus map of a content image. The defocus prediction module consists of a 1 channel expanded convolution layer, 7 residual blocks and a reconstruction layer. The channel expansion layer is responsible for expanding the 24-channel features into 48-channel features; the reconstruction layer uses a single convolutional layer and a sigmoid activation function to convert the 48-channel features into a single-channel defocus map. Since the defocus map is used for the fusion operation of the branch features of the stroke pyramid, the size of the defocus map needs to be the same as that of the branch features, and no upsampling operation is needed.

The previous style transfer approach does not use a variety of transfer strategies for the image subject and background, usually rigidly synthesizing a whole image with a single stroke. When the transfer texture adopts a monotonous and regular shape style, the boundary between the subject and the background in the generated image is weakened so that the viewer cannot see the content that the image wants to express. In this paper, we highlight the main area of the image by controlling the relationship between the degree of defocus blur and the stroke size in the spatial dimension. The reason for image defocusing is usually that the object is too far from the lens to focus.

The relationship between the degree of blur and the stroke size is based on the idea that the image clearly expresses itself. The higher the ambiguity of the region and with less semantic information, larger strokes should be used; the lower the ambiguity of the region and with more semantic information, smaller strokes should be used. This stroke fusion strategy can highlight the main area of the image and depict the content of the clear area in more detail. In this paper, the degree of ambiguity is divided into several levels. Different blur-level regions are synthesized using strokes of their corresponding sizes. There is a transition region between the adjacent regions of the fuzzy level, and the stroke size increases with increasing ambiguity in the transition region.

We use the defocus map obtained by defocus blur estimation as the stroke weight map *D*, and its range is [0,1]. Each element represents the size of the stroke in the spatial position, where 0 represents the larger stroke and 1 represents the smaller stroke. We enhance the contrast of the stroke weight map *D* to obtain the weight relationship between adjacent branches in the spatial dimension.

Let *W*_*i*_ be the weight relationship between the *i*-th branch and the i+1-th branch. The calculation formula of the weight relationship is:

$$\begin{array}{l} w_{i,j}=\left\{ \begin{array}{l} 0,d_{j}<u_{i}\\ \frac{d_{j}-u_{i}}{v_{i}-u_{i}},u_{i}\leq d_{j}<v_{i}\\ 1,d_{j}>v_{i} \end{array}\right.\\ i=1,\ldots n,j=1,\ldots N \end{array}$$ (4)

where *w*_*i*,*j*_ is the j-th weight in *W*_*i*_ and *d*_*j*_ is the j-th weight in the stroke weight graph *D*. *u*_*i*_,*v*_*i*_ are the corresponding transition thresholds.

Then, the weight relationship is converted into a set of soft masks, and each branch has its corresponding soft mask. The code value for the soft mask reflects the attention to the corresponding position of the branch feature in the spatial dimension. The calculation formula for the soft mask is as follows:

$$\left\{ \begin{array}{l} M_{1}=A-W_{1}\\ M_{1}=W_{i}-W_{i+1},i=2,\ldots ,n-1\\ M_{n}=W_{n} \end{array}\right.$$ (5)

where *M*_*i*_ is the soft mask corresponding to the i-th branch and A is the full 1-matrix with the same spatial dimension as the depth map.

Finally, the formula to calculate the soft mask gating function *G*_*i*_ of the i-th branch is given as follows:

$$G_{i}\left(D,F_{B_{i}}\right)=M_{i}⊗F_{B_{i}}$$ (6)

where *M*_*i*_ is the soft mask corresponding to the i-th branch, $F_{B_{i}}$ is the feature map output by branch $F_{B_{i}}$, and ⊗ represents the dot multiplication operation of the spatial dimension.

The feature maps output by all branches need to go through the soft mask gating function and accumulate. Then, that output goes through the decoder.

$$I_{o}=Dec(\sum_{i}G_{i}(D,F_{B_{i}}))$$ (7)

where *Dec* represents the decoder network, *I*_*0*_ represents the stylized image, and *G*_*i*_ represents the calculated result of the ith branch feature after going through the soft mask gating function.

#### 2.2.3 Loss function calculation

(1) Style transfer loss function

The loss function consists of three parts: content loss, style loss and total variation regularization loss. Content loss is used to preserve the semantic information in the content image. The calculation formula is as follows:

$$L_{c}=\sum_{l\in \{l_{c}\}}\left\|F^{l}(I_{c})-F^{l}(I_{o})\right\|$$ (8)

where *F*^*l*^(*I*_*o*_) and *F*^*l*^(*I*_*content*_) are the features extracted by the output image and the content image input to VGG-Net19 [35] in the *l* layer, respectively; the content extraction layer {*l*_*c*_} is set to {conv4 _ 1}.

The total variation regularization loss is used to improve the smoothness of the output image, and its implementation is consistent with the implementation method in [15]. Style loss is used to measure the style similarity between the style image and the output image. We use the style loss calculation method proposed in [13]. The calculation formula for the Gram matrix is as follows:

$$G(F^{l}{\left(I_{s}\right)}^{\prime })=[F^{l}{\left(I_{s}\right)}^{\prime }]{\left[F^{l}{\left(I_{s}\right)}^{\prime }\right]}^{T}$$ (9)

where $F^{l}{\left(I_{s}\right)}^{\prime }\in R^{C\times (H\times W)}$ is the feature matrix of feature map $F^{l}(I_{s})\in R^{C\times H\times W}$ after the reshaping operation.

Style loss consists of Gram style loss and mean standard deviation loss.

The calculation formula of Gram style loss is as follows:

$$L_{s1}=\sum_{l\in \{l_{s}\}}\left\|G(F^{l}{\left(R(I_{s},T_{B_{k}})\right)}^{\prime })-G(F^{l}{\left(I_{O}^{B_{k}}\right)}^{\prime })\right\|$$ (10)

The calculation formula of the mean standard deviation loss is as follows:

$$L_{s2}=\sum_{l\in \{l_{s}\}}\left[\left\|\mu (F^{l}(R(I_{s},T_{B_{k}})))-\mu (F^{l}(I_{O}^{B_{k}}))\|+\|\sigma (F^{l}(R(I_{s},T_{B_{k}})))-\sigma (F^{l}(I_{O}^{B_{k}}))\right\|\right]$$ (11)

where *I*_*s*_ represents the style image, $R(I_{s},T_{B_{k}})$ represents the style image scaled to $T_{B_{k}}$ resolution, and $F^{l}(R(I_{s},T_{B_{k}}))$ and $F^{l}(I_{o}^{B_{k}})$ represent the corresponding output image of branch *B*_*k*_ and the scaled style image input to the VGG-Net to extract the features in the *l* layer, respectively.

In this article, we set the style extraction layer with three branches {conv1 _ 2, conv2 _ 2, conv3 _ 3, conv4 _ 3}. The scaling resolution corresponding to each branch is set to x256, x512, x768respectively.

Finally, the total loss function is:

$$L=\lambda_{s1}L_{s1}+\lambda_{s2}L_{s2}+\lambda_{c}L_{c}+\lambda_{tv}L_{tv}$$ (12)

where $\lambda_{s1},\lambda_{s2},\lambda_{c},\lambda_{tv}$ are the superparameters of the Gram style loss, mean and standard deviation loss, content loss and tv loss, which are set as 1e5, 25, 1, 5 and 1e-7, respectively.

(2) Defocusing estimation loss function

The loss function consists of defocus loss and semantic loss.

The defocus loss is calculated from the MSE loss of the defocus predicted by the module and the validation defocus in the dataset. The semantic loss is calculated by the MSE loss of each layer feature extracted by VGG-Net from the original image and the prediction image.

The formula of the loss function is as follows:

$$L_{defocus}=\lambda_{1}\|I_{blur}-I_{O}\|+\lambda_{2}\|F^{l}(I_{blur})-F^{l}(I_{o})\|$$ (13)

where *I*_*blur*_ is the verification defocus map in the dataset, *I*_*o*_ is the defocus map output by the defocus estimation module, and *F*^*l*^(•) is the *l* layer feature extracted after the image is transmitted to VGG-Net. The extraction layer is {conv1 _ 2, conv2 _ 2, conv3 _ 3, conv4 _ 3}.

### 2.4 Experiment

#### 2.4.1 Experimental datasets

We use Microsoft’s Common Objects in Context (COCO)-2017 public dataset for image processing [36] to train the style transfer network. This dataset is a large-scale universal dataset for deep learning model training. It contains more than 330,000 images of objects of over 80 categories, and these objects possess abundant context information in real life. These images exhibit objects of different categories, such as people, vehicles, animals and furniture, as well as objects in different situations (e.g., an image may show a person in sportswear playing basketball on the court or a cat resting on the sofa). Compared with other datasets, the images contained in the COCO dataset contain more abundant context information, which makes them more challenging in style transfer tasks for speech information processing and more authoritative in calculating evaluation indicators. The images of the training set are scaled and cropped on the original basis and uniformly converted to 512x512 size. The SYNDOF dataset [34] is used, which contains artificially synthesized blurred images and corresponding defocus maps. The images and defocus maps of the training set are reduced by 4 times on the original basis.

#### 2.4.2 Training details

In this study, we use PyTorch to build the model and train it on a GeForce RTX 2080 Ti graphics card. The normalization operation is performed uniformly before the image is input into the feature extraction network. The averages of the input images are adjusted to [0.485, 0.456, 0.406], and the standard errors are adjusted to [0.229, 0.224, 0.225]. The number of samples contained in a single training batch is set to 2. For the dropout setting, we randomly set some feature values to 0 with a probability of 0.5 during training and set the skip threshold to 1e5 to ensure a skip over the current batch of training when the loss value fluctuates largely. In this study, the Adam optimizer is used for training, with parameters *β*_1_ = 0.5, *β*_2_ = 0.999, and *ε* = 1e-8.

The training in this paper is divided into two parts: the defocus estimation module and the stroke pyramid training.

For stroke pyramid training, the COCO-2017 dataset is used, and the learning rate is set to 1e-3. Since the training set is large enough, only 1 epoch is trained, and the learning rate attenuation is not set. To train the various stroke branches in a network, we adopt a progressive training strategy. Each training batch selects only one branch for training, and we poll the branch index so that the parameters of each branch can be updated. In addition, under the progressive training strategy, the training of the latter stroke branch benefits from the knowledge of the previously learned branch.

For defocus estimation module training, the SYNDOF depth of the field dataset is used for training, and the learning rate is set to 1e-4, which gradually decays to 1e-5 as the training epoch increases, with a total of 60 epochs trained.

#### 2.4.3 Experimental analysis

To better verify the use value of the model in this paper, we compare the model with other classical algorithms. From the training curve analysis, the verification of the effect of the residual block of the mixed cavity convolution, the comparison of the loss effect of the mean standard deviation, the comparison of the defocus adaptive and the single stroke, and ablation experiments were conducted in four parts.

## 3. Results and discussion

### 3.1 Contrast experiment

**Fig 5:**
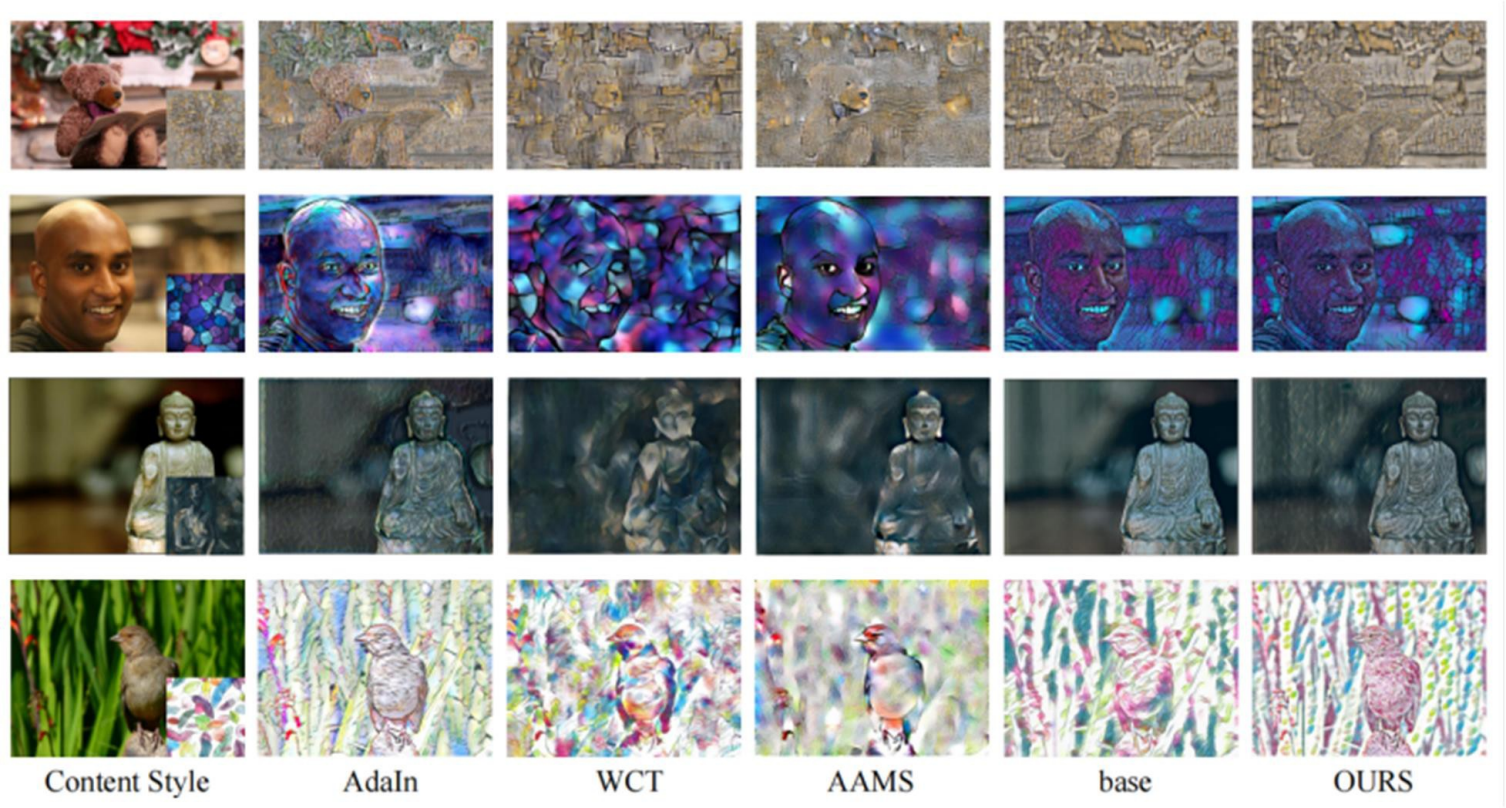
Comparison of results generated by the various methods.

To verify the effectiveness of the proposed method in this paper, the mainstream image style transfer methods AdaIn [22], WCT [10], AAMS [25] and model stroke pyramid [24] are used for effect comparison. The purpose of this method is to keep the content expression of the subject as much as possible when dealing with shallow depth of field images so that it does not integrate with the texture generated by the background. As shown in Fig 5, the main contour of the image generated by AdaIn is clear, but the color display is different from the style image. The image generated by WCT is better in color conversion, but the boundary between the subject and the background is not obvious; moreover, it is not easy to express its semantic information. The image generated by the method in this paper maintains a good semantic expression in a clear area, and the texture in the blurred area is rough so that it can more clearly show the content of the image focus. For example, in the first photo of a teddy bear, the teddy bear is the main content, which is the focus area of the image. In addition, behind the teddy bear is the background content, which is the defocused area of the image. After the content image is migrated, the gray‒yellow lattice style is obtained. The teddy bear area is expressed by a smaller lattice, and its detailed texture is displayed. The background area is expressed by a larger lattice to distinguish it from the main content, highlighting the semantic expression of the main content, and the transition between the main area and the background area is continuous and natural. Compared with those of the baseline model, the strokes of the images generated by the algorithm are more complete, and the shapes and lines are more similar to the style images. As in the second image, the image generated in this paper fully expresses the square block pattern, while the baseline model cannot describe the large strokes due to the lack of receptive field; thus, the purple color spots appear in the face area.

Since the style transfer results are difficult to evaluate by quantitative indicators, we use the peak signal-to-noise ratio (PSNR) and the structural similarity index (SSIM) to evaluate the antinoise ability of this method and the ability to maintain the semantic content images. In this paper, various methods are analyzed on the test set. The evaluation index uses the generated image and the content image to calculate the average value. The higher the two indices are, the better. As shown in Table 2, the proposed method has certain advantages in texture clarity and semantic information retention.

**Table 2:** Comparison of quantitative indices between the various methods.

	AdaIn		WCT		AAMS		base		OURS	
style image	PSNR	SSIM	PSNR	SSIM	PSNR	SSIM	PSNR	SSIM	PSNR	SSIM
composition	11.9	0.31	11.75	0.34	11.67	0.31	11.96	0.34	12.05	0.36
cubes	14.65	0.5	11.6	0.41	13.99	0.53	12.73	0.58	12.78	0.59
women	14.23	0.54	11.92	0.43	14.36	0.62	14.59	0.64	14.62	0.67
leaves	7.25	0.33	6.91	0.23	7.45	0.37	7.34	0.3	7.36	0.3

### 3.2 Ablation experiment

#### 3.2.1 Training curve comparison

**Fig 6:**
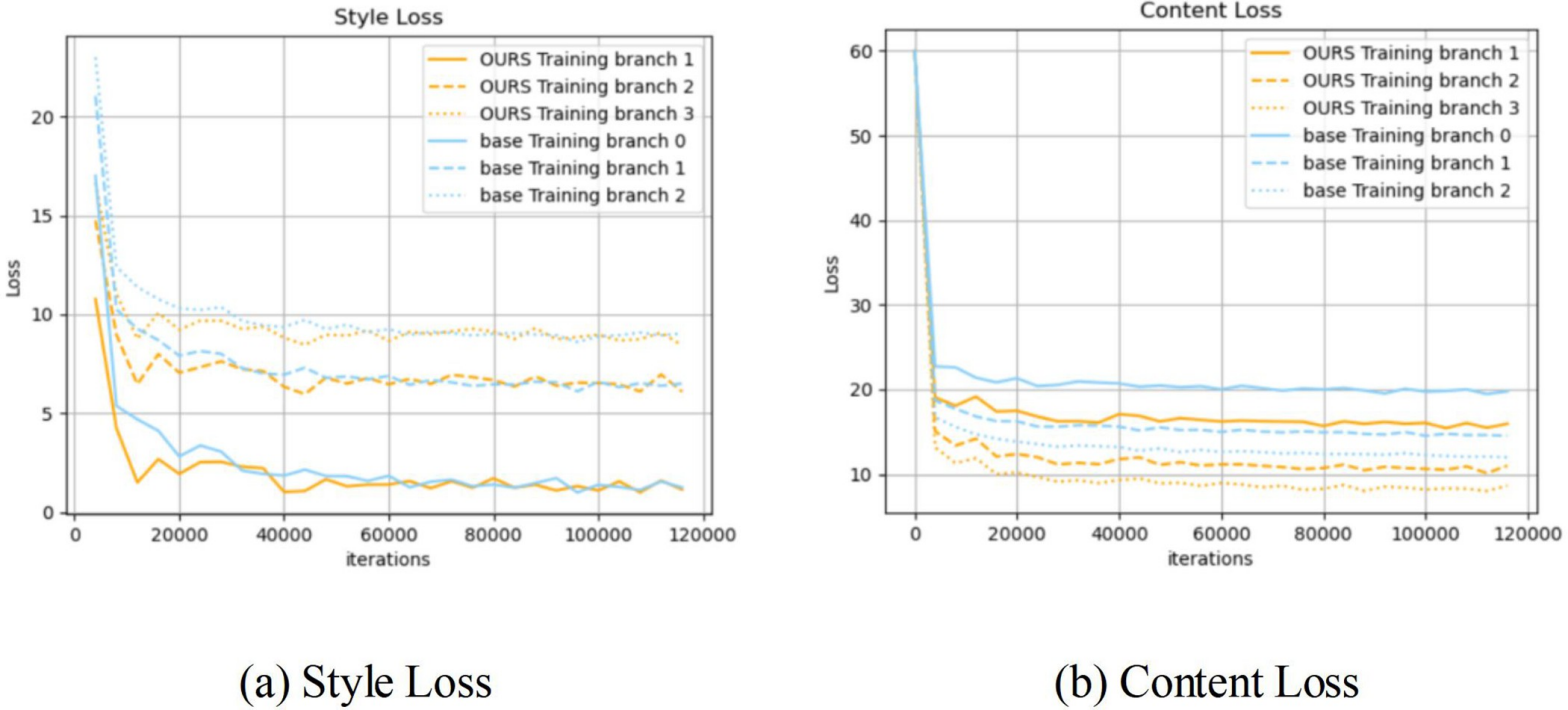
Comparison of the training curve between the network when using hybrid dilated convolution and the baseline model network.

To analyze the descent process of the loss function, we compared the training process of the baseline model network with the network with mixed dilated convolution. A test was performed on 4,000 samples per training, and the style loss and content loss of the results generated by the three branches were recorded. The recorded values were calculated as the average of the loss values of 4 style images and 100 content images. As shown in Fig 6, the style loss value calculated by each branch of the network with mixed dilated convolution decreased rapidly in the early stage of training, and the content loss was slightly lower than that of the baseline model, which indicates that the network training speed had a certain degree of improvement.

#### 3.2.2 Ablation experiment for the cavity convolution

Cavity convolution can expand the receptive field of the network so that the network can learn to depict larger strokes. To verify the effect of cavity convolution in this model, we selected several style images with larger strokes and compared the results of the network output by using ordinary residual blocks and the network output by using mixed cavity convolution residual blocks. To verify the ability of independent branches to depict strokes, we do not fuse the features of each branch and instead use the independent output results of each branch.

**Fig 7:**
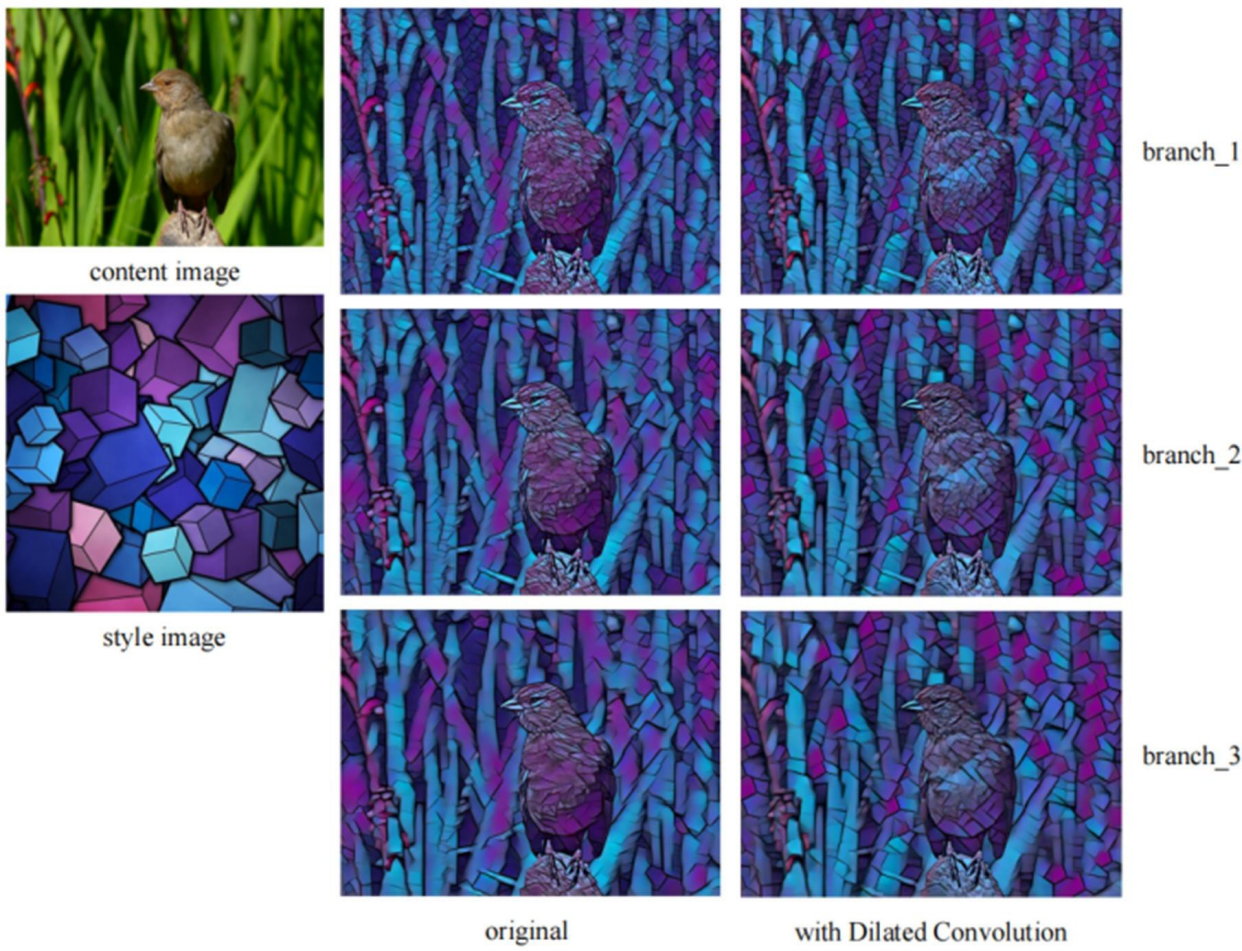
Comparison of the effect before and after the introduction of cavity convolution.

As shown in Fig 7, the image output by the network model when using the ordinary residual block is more complete in the strokes of the first two branches, while the last branch has no obvious shape features and does not draw a larger square block pattern. It can describe only the color and some lines of the style image; there are gradual color spots. The image output by the network model when using the mixed hole residual block can draw a large stroke well in the last branch, and the drawn block is more three-dimensional in the results generated by each branch.

#### 3.3.3 Ablation experiment for the mean standard deviation loss

To verify the influence of the mean standard deviation loss on the model in this paper, a style image with darker color was selected for comparative experiments. The network trained with mean standard deviation loss was compared with the network trained without mean standard deviation loss.

**Fig 8:**
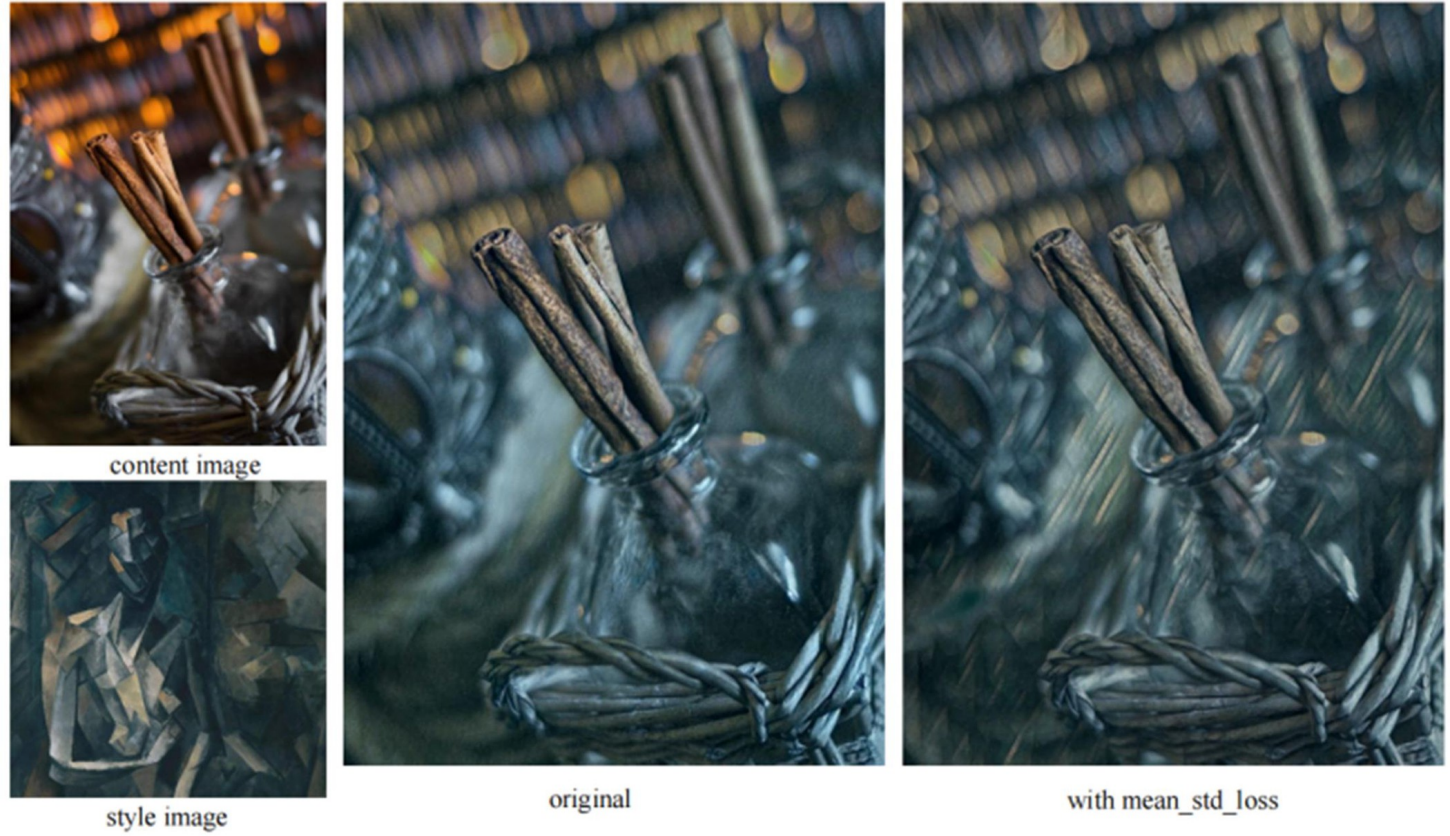
Comparison of effects before and after the introduction of mean standard deviation loss.

As shown in Fig 8, the image generated by using only the Gram matrix to calculate style similarity cannot effectively transfer the colorless style, and the effect is similar to color transfer. However, by introducing the mean standard deviation loss, the network can retain some structural information in the style image to a certain extent.

#### 3.3.4 Defocus adaptive ablation experiment

We compared the output results of the multi-stroke fused with defocus images approach with the results of the single stroke approach.

**Fig 9:**
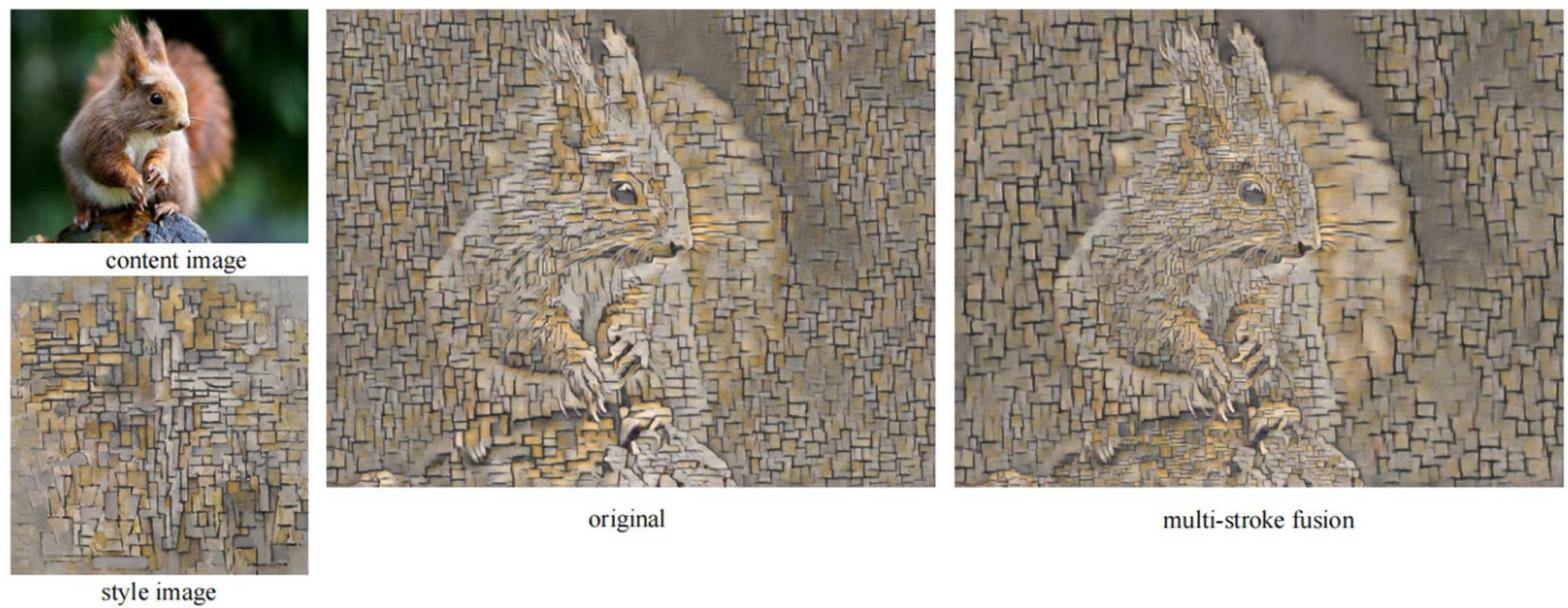
Comparison of the effects before and after the introduction of defocus adaptive multi-stroke feature fusion.

As shown in Fig 9, the use of multi-stroke fused with defocus images produces a three-dimensional effect. The texture displayed as the main area is more delicate and has smaller strokes. The objects displayed as the background area are depicted with larger strokes, and the painting style is bolder to highlight the semantic expression of the main area.

#### 3.3.5 Time performance test

To test the time performance of the framework proposed in this study, we reduce the size of the test samples from the COCO-2017 dataset, which contains 10,000 test samples, to 256, 512 and 1024, respectively, and then input them into the model. We calculate the time consumption of the style transfer module and defocus estimation module as well as the total time consumption of the entire framework. Table 3 summarizes the average time consumption of the module with seconds as the unit. Compared to the classical method proposed by Johnson et al. [15], our method uses convolutional layers with smaller channels, which can process large-size images faster. Although the processing speed of the style migration module involved in our study is slower than the baseline model [24] as a consequence of the increased network depth, the performance loss is within an acceptable range.

**Table 3:** Time performance table of each module under different image sizes (unit, second).

Image size	Style transfer module	Defocus estimation module	Total
256x256	0.0207	0.01194	0.0368
512x512	0.0234	0.01291	0.0398
1024x1024	0.0243	0.01495	0.0448

## 4. Conclusion

In this paper, an improved multi-stroke defocus adaptive style transfer based on a stroke pyramid is proposed. This method addresses the problem that image style transfer causes a certain loss of semantic information of the original image after giving the image a specific style. By introducing the stroke pyramid, the one-stop generation of stylized images with multiple stroke sizes is realized. By introducing the mixed dilated convolution, the receptive field of each branch network is expanded so that the network can learn the larger geometric structure features in the style image. By using the feedforward network to generate the defocus estimation map of the image in the multibranch fusion, the mixed expression of multiple strokes in the spatial dimension of the image is realized. The experimental results show that compared with that of the classical style transfer method, the texture of the image generated by our framework is more delicate in the clear area, the texture in the fuzzy area is softer, and the difference between the main area and the background area of the image is more significant. This approach makes it easier for viewers to understand the semantic content expressed by stylized images, and the PSNR and SSIM indicators are improved by 1.43 and 0.12 on average, respectively. Although the processing effect of the framework is better in subjective evaluation, the defocus estimation module uses fewer convolutional layers, and the defocus estimation is not accurate enough, so some missing image blocks are generated.

In the future, we will consider increasing the number of residual blocks in the defocus estimation module to 20, and we will add an attention mechanism to further improve image quality. Through these treatments, the model complexity can be increased, which enables the model to be more suitable for processing high-resolution images and complex scenes. In addition, we plan to increase the number of branches in the style migration network to 5 to obtain richer stroke and texture information and then determine whether the final effect can meet expectations according to the assessment of the results. For future prospects, the structure of arbitrary style transfer networks and multi-stroke style transfer may constitute one of the future development directions. These two methods can improve the flexibility of the network while maintaining high-quality image generation, allowing users to choose more styles and styles to generate images. In addition, these methods can greatly shorten the generation time and model size, thereby better adapting to practical application scenarios.
